# A Revised Hippocratic Oath for the Era of Digital Health

**DOI:** 10.2196/39177

**Published:** 2022-09-07

**Authors:** Bertalan Meskó, Brennan Spiegel

**Affiliations:** 1 The Medical Futurist Institute Budapest Hungary; 2 Cedars-Sinai Health System Los Angeles, CA United States

**Keywords:** hippocratic oath, digital health, eHealth, future, automation, ethics, viewpoint, medical perspective, physician perspective, ethical, digital divide, artificial intelligence, moral

## Abstract

Physicians have been taking the Hippocratic Oath for centuries. The Oath contains a set of ethical rules designed to guide physicians through their profession; it articulates a set of true north principles that govern the practice of medicine. The Hippocratic Oath has undergone several revisions, most notably in 1948 by the World Medical Association. However, in an era of rapid change in medicine, we believe it is time to update the Oath with modest but meaningful additions so that it optimally reflects 21st century health care. The rise of digital health has dramatically changed the practice of medicine in a way that could not have been easily predicted at the time Hippocrates outlined his ethical principles of medicine. Digital health is a broad term that encompasses use of digital devices and platforms, including electronic health records, patient-provider portals, mobile health apps, wearable biosensors, artificial intelligence, social media platforms, and medical extended reality, to improve the process and outcomes of health care delivery. These technologies have driven a cultural transformation in the delivery of care. We offer modest suggestions to help prompt discussion and contemplation about the current Oath and its relevancy to our changing times. Our suggestions are not meant to be a definitive set of final recommendations. Rather, we propose new text that bodies such as the World Medical Association might consider integrating into an updated Oath, just as previous changes were adopted to ensure the Oath remains relevant and impactful for all physicians and their patients.

## A Brief History of the Hippocratic Oath

Physicians have been taking the Hippocratic Oath for centuries [1]. The Oath contains a set of ethical rules designed to guide physicians through their profession; it articulates a set of true north principles that govern the practice of medicine.

Although there are no explicit penalties enforced upon physicians who break the Oath, adherence with its principles remains a time-honored tradition by practicing clinicians. The original Hippocratic Oath describes ideals that are timely and relevant even in the 21st century: to treat patients to the best of one’s ability; to preserve a patient’s privacy; and to faithfully teach the art of medicine to the next generation [2].

Most physicians believe the Oath still has relevance today, although viewpoints remain varied and there are few empirical studies that have formally evaluated sentiment about the Oath. In a non–peer-reviewed survey conducted in 2016, Medscape reported opinions about the modern relevance of Hippocrates’ pledge [3]. Nearly 3000 physicians and medical students responded. Reactions were markedly different, particularly when stratified by age. Of those under age 34 years, only 39% said the Oath was still meaningful, whereas 70% of respondents aged 65 years and older positively endorsed the pledge. Despite these varying views about the Oath, coupled with limited peer-reviewed data on this topic, most medical schools still ask their students to recite either the classic or modified form of the Oath.

The Hippocratic Oath has undergone several revisions, most notably in 1948 by the World Medical Association; this version is called the Declaration of Geneva [4]. Changes included removing lines such as “My colleagues will be my sisters and brothers” and added lines such as “I will respect the autonomy and dignity of my patient.” However, in an era of rapid change in medicine, we believe it is time to update the Oath with modest but meaningful additions so that it optimally reflects 21st century health care.

## Why a Revised Hippocratic Oath Is Warranted in the Era of Digital Health

The rise of digital health has dramatically changed the practice of medicine in a way that could not have been easily predicted at the time Hippocrates outlined his ethical principles of medicine. Digital health is a broad term that encompasses use of digital devices and platforms, including electronic health records, patient-provider portals, mobile health apps, wearable biosensors, artificial intelligence (AI), social media platforms, and medical extended reality, to improve the process and outcomes of health care delivery. These technologies have driven a cultural transformation in the delivery of care [5].

Remote patient monitoring, for example, affords patients and doctors a more complete and accurate picture of disease progression outside the walls of a hospital, clinic, or research center. The data from mobile technologies can now be shared between patient and provider, allowing greater collaboration, stronger therapeutic partnerships, enhanced shared decision-making, and an increasing shift to preventive and proactive care in lieu of reactive care.

Extended reality technologies, such as virtual reality and augmented reality, provide opportunities to go beyond the traditional exam room and introduce new ways of blending behavioral and psychosocial care with traditional biomedical care [6]. AI is massively expanding our ability to diagnose and treat patients, but simultaneously raising significant ethical debates about the potential for misuse of powerful and potentially biased algorithms [7]. Social media platforms have become a digital town hall for all manner of health care information exchange, further democratizing access to information historically under the sole purview of physicians.

In short, the cultural transformation enabled by digital health is rapidly changing the practice of medicine from a tradition of physician-driven decisions based on limited, institutionally owned data, to shared decision-making based on expansive data across platforms, owned and shared by patients, that reflects biopsychosocial well-being across broad disease trajectories and illness experiences along a range of geographies, demographics, and sociocultural communities. In light of this transformation, we believe it is warranted to modestly update the Hippocratic Oath so it optimally reflects 21st century medicine.

## Proposed Revisions to the Hippocratic Oath

In the context of changes produced by advances in digital health, we suggest that the following principles should be reflected within a modernized Hippocratic Oath, presented in order of the current Oath’s text.

### Recognize a Broader Origin of Scientific Gains in Medicine

The Hippocratic Oath entreats physicians to “respect the hard-won scientific gains of those physicians on whose steps I walk.” This statement suggests that research is conducted by physicians only, when in fact medical advances originate from many stakeholders beyond physicians. In the era of digital health and democratized care, research arises not only from physicians and nonclinical researchers, but also from patients who both contribute their own data and meaningfully participate in research through patient-centered models such as those supported by the Patient-Centered Outcomes Research Institute [8]. We suggest the following edits, shown in italics:

- I will respect the hard-won scientific gains of those physicians, *researchers, and patients* in whose steps I walk, and gladly share such knowledge as is mine with those who are to follow.

### Acknowledge Both “Sick Care” and Preventive “Health Care”

The Oath focuses on treating the sick but is silent on the role of preventive medicine for the well, yet modern medicine emphasizes the importance of preventive care across physical, mental, and social realms of health, not just reactive “sick care” for the ill. Advances in digital health place an emphasis on predictive analytics using remotely collected data, and precision medicine aims to identify early signs of disease to inform timely preventive care. Given these forces that are shifting medical delivery from reactive “sick care” to preventive “health care,” we propose the following simple addition to the Oath, shown in italics:

- I will apply, for the benefit of *the healthy and* the sick, all measures [that] are required, avoiding those twin traps of overtreatment and therapeutic nihilism.

### Reflect the Intrinsic Use of Digital Technology in the Practice of Medicine

Considering the massive advances in technology, coupled with the reality that technology now plays an everyday role in the delivery of health care, we believe the Hippocratic Oath should reflect the foundational role of digital health in patient care. As advances in AI, robotics, virtual reality, mobile health apps, wearable biosensors, and portable diagnostic devices continue to expand, we believe the Oath should acknowledge the growing and permanent impact these technologies now exert on care delivery, as follows:

- I will remember that there is an art to medicine as well as science, and that warmth, sympathy, and understanding may outweigh the surgeon’s knife, the chemist’s drug, *or the programmer’s algorithm.*

### Validate the Patient Role in an Equal-Level Partnership With Their Physician

The Hippocratic Oath encourages physicians to say, “I know not” when they are unsure how to treat a patient, and to “call in my colleagues when the skills of another are needed for a patient’s recovery.” These are laudable sentiments that any clinician should follow. However, the Oath should ideally acknowledge that patients, too, can help with diagnosis and treatment.

Although the tradition of medicine reinforces a hierarchical patient-doctor relationship driven by information asymmetry, patients now have expansive access to credible information about biomedical sciences, increasingly generate their own biometric health data through wearable biosensors, and monitor their own psychometric scores through apps; these data sources are now part of clinical practice.

Although physicians have more experience prescribing treatments and monitoring a wide range of diseases than nonphysician patients, the Oath should recognize that patients are the experts of their personal illness experience. When engaged collaboratively by their physicians, patients can deliver meaningful insights that shape diagnostic and care plans. In light of this paradigm shift that has accelerated in the era of digital health, we suggest the following italicized additions to the Oath:

- *I will treat my patients in an equal-level partnership, and* I will not be ashamed to say ‘I know not,’ nor will I fail to call in my colleagues when the skills of another are needed for a patient’s recovery.

### Address Data Privacy

Respecting patients’ privacy is a primary passage in the Oath. However, the concept of privacy now extends beyond safekeeping conversations to guarding the “big data” generated in the care of every patient in modern health care. We suggest the following addition:

- I will respect the privacy of my patients *and their data*, for their problems are not disclosed to me that the world may know.

### Emphasize the Primacy of Treating Patients, Not Their Data

The explosion in big data surrounding health care is transforming how doctors care for and interact with their patients. AI in particular has a vast potential to automate processes in health care and potentially overtake certain roles and responsibilities normally filled by clinicians. Nonetheless, physician must always remain focused on their patients, including their personal stories and their biopsychosocial well-being beyond their digital fingerprints and big data analytics. AI will never replace medical professionals, although physicians who embrace AI may eventually replace those who do not. We propose the following additions to the Oath to reflect these considerations:

- I will remember that I do not treat a fever chart, a cancerous growth, *a data point, or an algorithm’s suggestion,* but a human being.

## Conclusions

The Hippocratic Oath remains an important pledge that modern physicians should continue to honor. The Oath outlines principles that remain relevant in the 21st century. However, advances in digital health science and technology have catalyzed a cultural revolution in the delivery of health care.

We believe that it is now justified to modify the Hippocratic Oath—even if modestly—to reflect the digital health revolution, advances in patient empowerment, and the evolving role of technology in the everyday practice of medicine.

We offer these modest suggestions to help prompt discussion and contemplation about the current Oath and its relevancy to our changing times. Our suggestions are not meant to be a definitive set of final recommendations. Rather, we propose new text that bodies such as the World Medical Association might consider integrating into an updated Oath, just as previous changes were adopted to ensure the Oath remains relevant and impactful for all physicians and their patients (Multimedia Appendix 1).

## Appendix

Multimedia Appendix 1 Revised Hippocratic Oath.

## Abbreviations

AI: artificial intelligence
