# Supplementary material for: A Revised Hippocratic Oath for the Era of Digital Health
Source: J Med Internet Res. 2022 Sep 7;24(9):e39177. doi: 10.2196/39177 (PMC9497634; doi:10.2196/39177)
Supplement: Multimedia Appendix 1 [file jmir_v24i9e39177_app1.docx]

**Multimedia Appendix 1. Revised Hippocratic Oath.**

The revised Hippocratic Oath with the changes in italic.

**I swear to fulfill, to the best of my ability and judgment, this covenant:**

“I will respect the hard-won scientific gains of those physicians, *researchers, and patients* in whose steps I walk, and gladly share such knowledge as is mine with those who are to follow.”

“I will apply, for the benefit of *the healthy* and the sick, all measures [that] are required, avoiding those twin traps of overtreatment and therapeutic nihilism.”

“I will remember that there is an art to medicine as well as science, and that warmth, sympathy, and understanding may outweigh the surgeon’s knife, the chemist’s drug, *or the programmer’s algorithm.*

“*I will treat my patients in an equal-level partnership, and* I will not be ashamed to say “I know not,*”* nor will I fail to call in my colleagues when the skills of another are needed for a patient’s recovery.”

“I will respect the privacy of my patients *and their data*, for their problems are not disclosed to me that the world may know.”

**Most especially must I tread with care in matters of life and death. If it is given me to save a life, all thanks. But it may also be within my power to take a life; this awesome responsibility must be faced with great humbleness and awareness of my own frailty. Above all, I must not play at God.**

“I will remember that I do not treat a fever chart, a cancerous growth, *a data point,* *or an algorithm’s suggestion* but a human being.”

**My responsibility includes these related problems, if I am to care adequately for the sick.**

**I will remember that I remain a member of society, with special obligations to all my fellow human beings, those sound of mind and body as well as the infirm.**

**If I do not violate this oath, may I enjoy life and art, respected while I live and remembered with affection thereafter. May I always act so as to preserve the finest traditions of my calling and may I long experience the joy of healing those who seek my help.**
